# Efficacy of Low-Level Laser Therapy for Tinnitus: A Systematic Review with Meta-Analysis and Trial Sequential Analysis

**DOI:** 10.3390/brainsci10120931

**Published:** 2020-12-02

**Authors:** Chih-Hao Chen, Chii-Yuan Huang, Chun-Yu Chang, Yen-Fu Cheng

**Affiliations:** 1 Department of Otolaryngology-Head and Neck Surgery, Taipei Veterans General Hospital, Taipei 112, Taiwan; michaelchen808@gmail.com (C.-H.C.); dopod0635@gmail.com (C.-Y.H.); 2 Faculty of Medicine, National Yang-Ming University, Taipei 112, Taiwan; 3 Department of Anesthesiology, Taipei Tzu Chi Hospital, Buddhist Tzu Chi Medical Foundation, New Taipei City 231, Taiwan; paulchang1231@gmail.com; 4 Department of Medical Research, Taipei Veterans General Hospital, Taipei 112, Taiwan; 5 Institute of Brain Science, National Yang-Ming University, Taipei 112, Taiwan

**Keywords:** low level laser, tinnitus, meta-analysis, trial sequential analysis

## Abstract

Study Objective: Tinnitus is a common disorder characterized by sound in the ear in the absence of external or internal stimuli. Low-level laser therapy (LLLT) was discovered enhancing tissue repair via increasing the blood microcirculation and cell proliferation in 1960s. In the last two decades, LLLT delivered to the cochlea has frequently been used to reduce the severity of tinnitus. However, whether LLLT effectively attenuates the severity of tinnitus remains controversial. We aimed to evaluate the efficacy of low-level laser therapy on adult patients with complaints of tinnitus. Design: Systematic review and meta-analysis with trial sequential analysis. Interventions: Low-level laser therapy (LLLT). Measurements: Tinnitus Handicap Inventory (THI) score; improvement rates of the visual analog scale (VAS), verbal rating scale (VRS) and numeric rating scale (NRS) scores. Methods: We searched PubMed, Embase, Scopus, Web of Science, and the Cochrane Library from inception through 17 September 2020. Randomized control trials that involved adult patients with complaints of tinnitus, compared LLLT to a placebo and provided sufficient information for meta-analysis were considered eligible. Main Results: Overall, 11 studies involving 670 patients were included. No significant difference in the overall effect according to the THI score (mean difference (MD), −2.85; 95% CI, −8.99 to 3.28; *p* = 0.362; *I*^2^ = 0%) and the rating scale score improvement rate (risk ratio (RR), 1.35; 95% CI, 0.81 to 2.27; *p* = 0.250; *I*^2^ = 67%) was demonstrated between patients receiving LLLT and those receiving a placebo. None of the subgroup analyses showed significant differences, regardless of underlying sensorineural hearing loss, the number of irradiation sessions or the wavelength used. Conclusions: Our meta-analysis suggests that the value of LLLT in controlling the severity of tinnitus remains unclear, in part due to the relatively small number of patients and underlying heterogeneity. More large-scale investigations of LLLT for tinnitus related to inner ear disease are required to further elucidate the therapeutic effects.

## 1. Introduction

Tinnitus is a common disorder characterized by sound in the ear in the absence of external or internal stimuli. Although there are numerous etiologies responsible for tinnitus, idiopathic tinnitus still accounts for most cases [1]. Persistent tinnitus can cause devastating disturbances and morbidities at the psychological and socioprofessional levels [2,3,4,5]; therefore, many attempts have been made to relieve tinnitus. However, none of the symptomatic treatments have resulted in significant and lasting improvement of tinnitus.

In contrast to high power lasers that are used to cut or destroy tissue, low-level laser therapy (LLLT) applies lasers with lower power to the surface of the body. LLLT acts by increasing blood microcirculation through sympathetic neural inhibition, prompting an increase in cell proliferation and enhancing adenosine triphosphate (ATP) synthesis in mitochondria. Together, it speeds up the repair and decreases the damage of cells and tissue [6,7,8]. However, it was not until Moon et al. [9] assessed the safety of LLLT in an animal model that a laser power of less than 200 mW could be safely administered to the tympanic membrane without adverse effects such as edema, vascular congestion and inflammation. Since the effectiveness of LLLT was shown for several conditions, including pain for rheumatoid arthritis [10], osteoarthritis [11], chronic low back pain [12], acute and chronic neck pain [13] and tendinopathy [14], LLLT has been considered a potential treatment for tinnitus in the past two decades.

However, the therapeutic efficacy of LLLT is still controversial, as only some studies have shown positive results. The differences may result from inconsistencies in several factors. First, as a higher wavelength laser would deliver a large amount of irradiance through greater penetration [15,16,17], different wavelength settings might affect the efficacy of LLLT. Second, LLLT delivered to the cochlea via the transmastoidal route is expected to be greatly absorbed by temporal bone, leading to therapeutically insufficient doses of irradiation. Trans-meatal delivery of LLLT, on the other hand, shows more irradiation penetration since a less solid structure hinders irradiation [15]. The delivery route may also influence the results of LLLT. Finally, different irradiance dose exposures could also account for the different results [17]. In addition, different types of measurements have been used in the evaluation of tinnitus severity, including self-reported questionnaires (e.g., Tinnitus Handicap Inventory (THI), Tinnitus Severity Index (TSI)) and rating scales (e.g., visual analog scale (VAS), verbal rating scale (VRS), numeric rating scale (NRS)). Measurement inconsistencies between studies further hinder authors and clinicians from making comparisons among studies.

In light of these issues, the present study aims to systematically review the current literature on LLLT. To explore the true effect of LLLT and the influence of concurrent factors, we identified randomized controlled trials (RCTs) and sought to evaluate the efficacy of LLLT in adult patients with tinnitus via meta-analysis.

## 2. Materials and Methods

### 2.1. Study Design

The present study is a meta-analysis and systematic review of randomized control trials. The primary objective of this study is to explore the effects of LLLT on patients with complaints of tinnitus. This study follows the Preferred Reporting Items for Systematic Review and Meta-analysis (PRISMA) statement [18]. This study is also registered in the International Prospective Register of Systematic Reviews (CRD42020209916).

### 2.2. Search Strategy

From their inception through 17 September 2020, databases including the Cochrane Library, PubMed, Embase, Web of Science, and Scopus were searched by two authors (C.-H. Chen and C.-Y. Chang). We used subject headings (Medical Subject Headings (MeSH) terms in the Cochrane Library and PubMed, and Emtree terms in Embase) and search field tags of title, abstract and keywords to facilitate searching. The Boolean operator “OR” was used to cover similar concepts, whereas “AND” was used to identify where different concepts intersect. We also consolidated MeSH and text words to create two subsets of citations: one including studies of lasers (“laser”, “pulsed laser”, “continuous wave laser”) and the second including studies on tinnitus (“tinnitus”, “subjective tinnitus”, “objective tinnitus”). Supplementary Table S1 shows the detailed search strategy. The identified records were screened by title, abstract, and keywords. Records with potential eligibility were then obtained and subjected to a full-text review. To identify additional studies, a manual search of the reference lists of the included studies was conducted.

### 2.3. Eligibility Criteria

Two reviewers (C.-H. Chen and C.-Y. Chang) selected the studies that met all of the conditions of the following criteria: (a) the study was an RCT involving patients undergoing LLLT for tinnitus; (b) the study compared LLLT with a placebo and reported an outcome of interest (i.e., THI); and (c) the study provided adequate information to calculate the effect estimates for meta-analysis. We did not exclude studies based on publication date, language, or geographical area. When there were discrepancies regarding the inclusion of a study, a third author (Y.-F. Cheng) would provide consensus or discussion.

### 2.4. Risk of Bias Assessment

The revised Cochrane Risk of Bias Tool 2 was used to assess the methodological quality of the included studies. Disagreements between the two reviewers were resolved through either discussion or consensus with a third reviewer (Y.-F. Cheng).

### 2.5. Data Extraction

Two reviewers (C.-H. Chen and C.-Y. Chang) extracted datasets from the eligible studies. The extracted information included author’s name, publication year, country, number and mean age of patients, laser type and intensity, scale used for tinnitus measurement, timing of the measurement, adverse events that had been assessed and reported, and effect estimates

### 2.6. Statistical Analysis

The effect estimate for the Tinnitus Handicap Inventory (THI) was the mean difference (MD) and that for the improvement rate estimated by the rating scale scores was the risk ratio (RR). The MD and 95% confidence interval (CI) were calculated directly from data reported in tables or the main text. The pooled MD and RR were calculated using the inverse variance method. Based on the assumption that ethnicity, country, underlying disease and age differences existed in the patient populations across studies, random-effects meta-analysis models with the DerSimonian–Laird estimator were selected to account for a second source of error in addition to the sampling error that is expected with a fixed-effect model. The Cochran Q statistic and the *I*^2^ statistic were used to assess statistical heterogeneity. The heterogeneity was considered low, moderate and high for an *I*^2^ of <50%, 50–74%, and ≥75%, respectively [19].

Subgroup analyses were conducted to explore the influence of underlying disorders, including sensorineural hearing loss (SNHL) and idiopathic tinnitus, the length of the irradiation session and different wavelength settings, on the pooled effect estimates, as these factors might cause differences in the effects of LLLT [1,15,16]. In the influence analysis of the THI score after intervention, the pooled point estimates after omitting each included study one at a time lay within the 95% CI of the overall pooled results. Similarly, the influence analysis of the scale score improvement rate after intervention revealed the same results.

To obtain a conclusive meta-analysis and evaluate if the obtained results could have been type I or type II errors caused by sparse data and lack of power, the diversity-adjusted required information size (RIS) and trial sequential monitoring boundaries were calculated through trial sequential analysis (TSA) [20]. The models for all outcomes were based on an alpha of 5% and a power of 80%. All statistical analyses were performed using R version 4.0.2 with the “dmetar”, “meta”, and “metafor” packages [21]. TSA software version 0.9.5.10 Beta was used to perform the TSA. Statistical results were considered significant when there was a *p*-value < 0.05 [22].

## 3. Results

### 3.1. Study Identification and Selection

**Figure 1:**
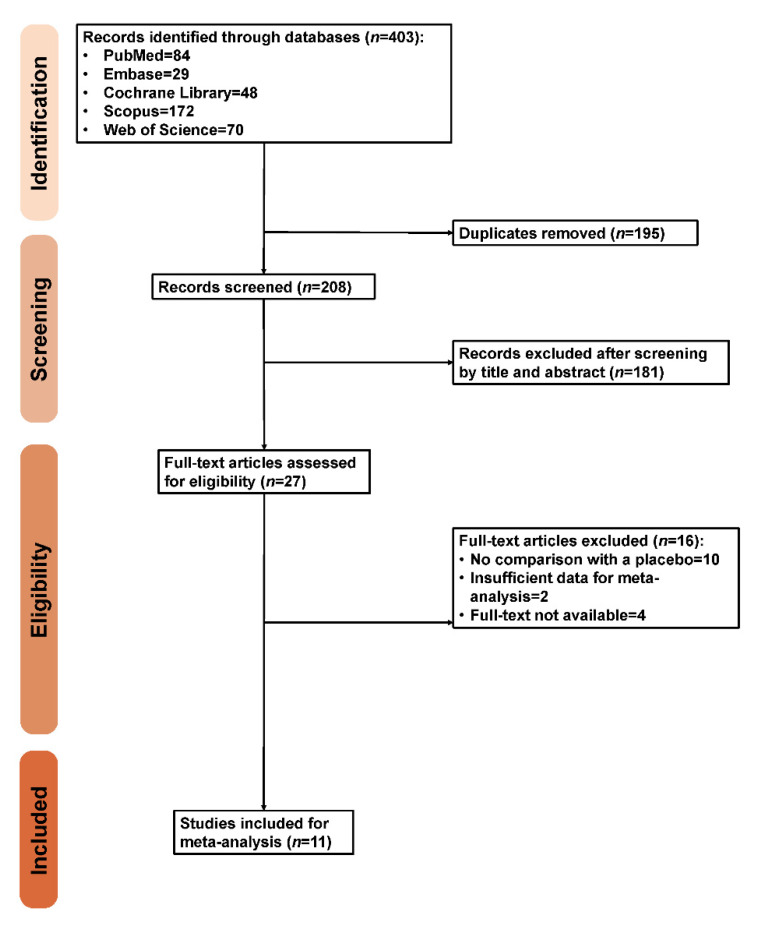
Preferred Reporting Items for Systematic Review and Meta-analysis (PRISMA) flow diagram.

We identified 403 records in five databases, namely, the Cochrane Library (*n* = 48), PubMed (*n* = 84), Scopus (*n* = 172), Embase (*n* = 29) and Web of Science (*n* = 70). After removal of 195 duplicates, we screened the remaining studies for eligibility. Based on irrelevance, 181 studies in total were excluded after reviewing the title and abstract. Twenty-seven studies entered the full-text review. Sixteen studies were then excluded due to a lack of comparison with a placebo, insufficient data for meta-analysis and unavailability of the full text. As a result, 11 studies containing 670 patients were included. Figure 1 presents the exhaustive PRISMA flow diagram.

### 3.2. Study Characteristics and Risk of Bias Assessment

The study characteristics are presented in Table 1. Two studies enrolled diseased ears as samples [23,24]. One study used a low laser device with an intensity of 100 mW and a wavelength of 650 nm for ten sessions [1], one used the device with an intensity of 5 mV and a wavelength of 650 nm for twenty sessions [25], one used the device with an intensity of 5 mW and a wavelength of 650 nm for twenty sessions [26], one used the device with an intensity of 5 mW and a wavelength of 650 nm for seventy sessions [27], two used the device with an intensity of 5 mW and a wavelength of 650 nm for ninety sessions [28,29], one used the device with an intensity of 5 mW and a wavelength of 650 nm for seven sessions [23], one used the device with an intensity of 67 mW and a wavelength of 830 nm for twelve sessions [30], one used the device with an intensity of 60 mW and a wavelength of 810 nm for four sessions [24], and one used the device with an intensity of 50 mW and a wavelength of 830 nm for fifteen sessions [31]. Five studies enrolled patients with SNHL [1,24,26,28,32], whereas four studies included patients with idiopathic tinnitus [25,27,30,31], and the other two studies included patients with mixed tinnitus involving hearing loss and idiopathic tinnitus [23,29]. Nine of the included studies enrolled patients with tinnitus lasting for more than 6 months [23,25,26,27,28,29,30,31,32], while one study enrolled patients with tinnitus lasting for more than three months [1] and the other one did not report the duration [24]. Ten of the included studies performed sham laser as placebo intervention [1,23,24,25,26,27,28,29,31,32], while the other one did not state the intervention in control group [30]. Five of the included studies performed laser unilaterally [1,23,25,28,31], while the other studies did not further report the treatment laterality [24,26,27,29,30,32]. Tinnitus severity and improvement were measured immediately after intervention in eight studies [1,25,26,27,28,29,31,32], while one study measured tinnitus severity two weeks after the intervention [23], and two measured tinnitus severity one week after the intervention [24,30]. The THI score was used in five studies [1,28,29,30,31], and rating scales were used to evaluate whether patients experienced improvement in the loudness of tinnitus in six studies [23,24,25,26,27,32]. Adverse event observations were reported qualitatively in four studies [1,23,24,30]. More detailed information regarding the timing of the measurements, study results, laser modalities used for the interventions, measurement scales used and reported adverse events are presented in Table 1. Moreover, Supplementary Figure S1 presents the risk of bias assessment for each included study.

### 3.3. Outcomes

#### 3.3.1. THI Scores after LLLT

**Figure 2:**
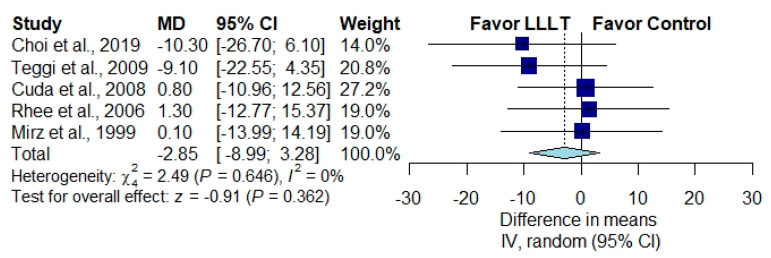
Overall effect of low-level laser therapy (LLLT) as measured by the THI score [1,28,29,30,31].

The THI scores were measured after LLLT and were reported in five studies [1,28,29,30,31] (Figure 2). Overall, the pooled THI level was lower in patients receiving low laser therapy than in those receiving a placebo, but this result did not reach statistical significance (MD, −2.85; 95% CI, −8.99 to 3.28; *p* = 0.362; *I*^2^ = 0%).

#### 3.3.2. Improvement Rate According to Rating Scale Scores

**Figure 3:**
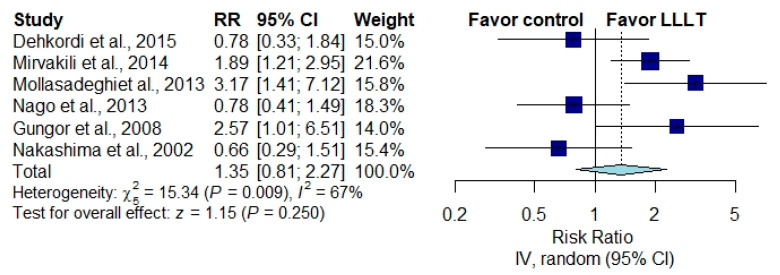
Overall effect of LLLT as measured by the rating scale score improvement rate [23,24,25,26,27,32].

The pooled results of six studies for improvement in the loudness of tinnitus showed no significant difference in the improvement rate between patients receiving LLLT and those receiving a placebo [23,24,25,26,27,32] (Figure 3) (RR, 1.35; 95% CI, 0.81 to 2.27; *p* = 0.250; *I*^2^ = 67%).

#### 3.3.3. Subgroup Analysis in Patients with SNHL or Idiopathic Tinnitus

**Figure 4:**
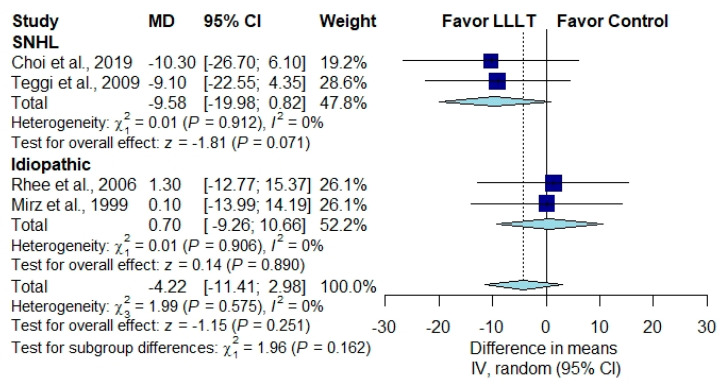
Subgroup analysis of THI scores in patients with SNHL and patients with idiopathic tinnitus [1,28,30,31].

**Figure 5:**
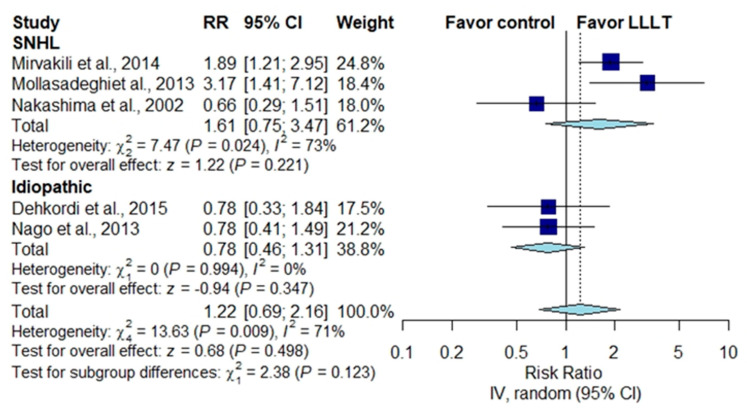
Subgroup analysis of rating scale score improvement in patients with SNHL and patients with idiopathic tinnitus [23,24,25,26,31].

In patients with SNHL, the pooled THI score was not significantly different between the irradiation group and the placebo group in patients with SNHL [1,28] (Figure 4) (MD, −9.58; 95% CI, −19.98 to 0.82; *p* = 0.071; *I*^2^ = 0%) or in patients with idiopathic tinnitus [30,31] (Figure 4) (MD, −9.58; 95% CI, −19.98 to 0.82; *p* = 0.071; *I*^2^ = 0%). The pooled results for improvement in the loudness of tinnitus demonstrated no significant difference between patients receiving LLLT and those receiving a placebo in patients with SNHL [24,26,32] (Figure 5) (RR, 1.61; 95% CI, 0.75 to 3.47; *p* = 0.221; *I*^2^ = 73%) or in patients with idiopathic tinnitus [25,27] (Figure 5) (RR, 1.22; 95% CI, 0. to 2.16; *p* = 0.347; *I*^2^ = 71%).

#### 3.3.4. Subgroup Analysis According to the Number of Irradiation Sessions

**Figure 6:**
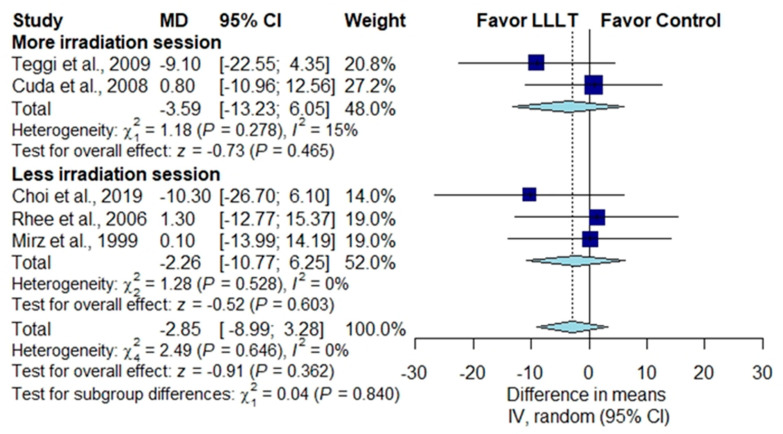
Subgroup analysis of THI scores according to the number of irradiation sessions [1,28,29,30,31].

**Figure 7:**
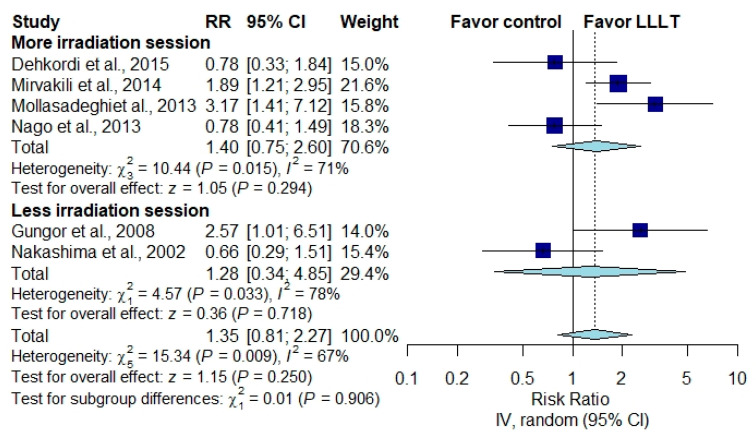
Subgroup analysis of rating scale score improvement rates according to the number of irradiation sessions [23,24,25,26,29,31].

In studies in which effect estimates were measured by THI score, two studies with more than the median number of irradiation sessions showed no significant difference between the LLLT group and the placebo group [28,29] (Figure 6) (MD, −3.59; 95% CI, −13.23 to 6.05; *p* = 0.465; *I*^2^ = 15%), while another three studies with fewer than the median number of sessions showed no significant difference between the two groups [1,30,31] (Figure 6) (MD, −2.26; 95% CI, −10.77 to 6.25; *p* = 0.362; *I*^2^ = 0%). In studies in which effect estimates were measured by the improvement rate of the rating scale scores, four studies with more than the median number of irradiation sessions revealed no significant difference between the two groups [25,26,27,32] (Figure 7) (RR, 1.40; 95% CI, 0.75 to 2.60; *p* = 0.294; *I*^2^ = 71%), neither did another two studies with fewer than the median number of sessions [23,24] (Figure 7) (RR, 1.28; 95% CI, 0.34 to 4.85; *p* = 0.718; *I*^2^ = 78%).

#### 3.3.5. Subgroup Analysis According to Wavelength Setting

**Figure 8:**
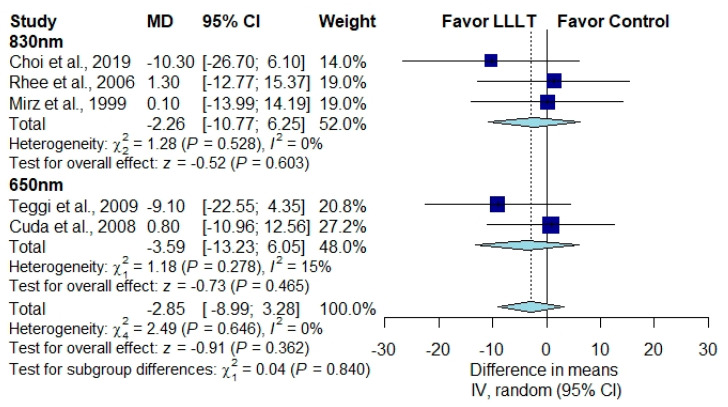
Subgroup analysis according to wavelength setting [1,28,29,30,31].

Three studies used a wavelength setting of 830 nm, and the pooled effect estimate demonstrated no significant difference between the LLLT group and the placebo group [1,30,31] (Figure 8) (MD, −2.26; 95% CI, −10.77 to 6.25; *p* = 0.362; *I*^2^ = 0%); similarly, two studies with a wavelength setting of 650 nm showed no significant difference between the two groups [28,29] (Figure 8) (MD, −3.59; 95% CI, −13.23 to 6.05; *p* = 0.465; *I*^2^ = 15%).

### 3.4. Influence Analysis

In the influence analysis, the pooled point estimates after excluding every study one by one were contained within the 95% CI of the overall pooled results for these outcomes (Supplementary Figures S2 and S3).

### 3.5. Trial Sequential Analysis

None of the cumulative Z-curves surpassed the traditional significance boundary or the sequential monitoring boundaries for the adjusted significance threshold in favor of LLLT in the TSA of the overall effect or in the subgroup TSAs (Supplementary Figures S4–S15).

## 4. Discussion

**Figure 9:**
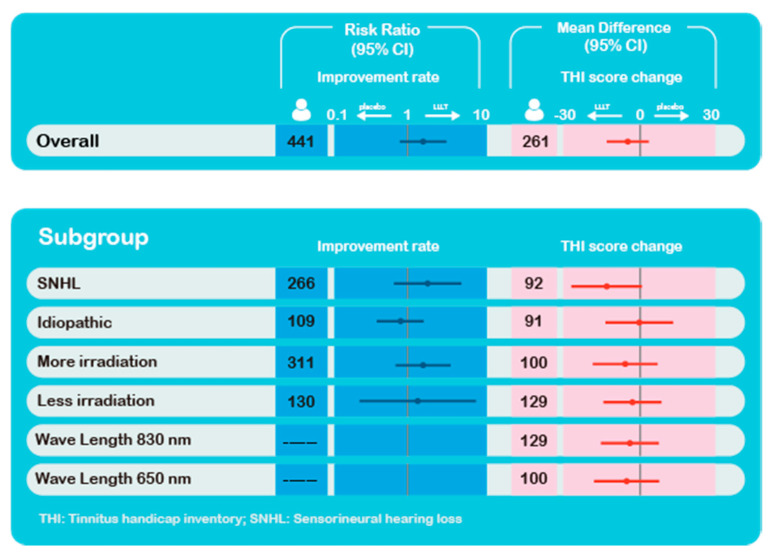
Visual summary of the results.

In this study, we analyzed the efficacy of LLLT for patients with tinnitus using a meta-analysis to obtain a meaningful conclusion. For the five studies evaluating tinnitus improvement after intervention by THI score [1,28,29,30,31], the pooled effect estimate did not show a significant difference. Moreover, the pooled effect estimate did not reveal a difference in the improvement rate of the loudness of tinnitus within six studies [23,24,25,26,27,32]. To explore whether LLLT demonstrated different efficacy for tinnitus in patients with SNHL and idiopathic tinnitus, we conducted a subgroup analysis using nine studies, while the subgroup analysis of the influence of different wavelength and irradiation session settings was performed using all studies. None of the pooled estimates of the subgroup analysis demonstrated a significant difference between the low-level laser group and the control group. A visual summary of the results is presented in Figure 9.

The theoretical mechanism behind the therapy is assumed to involve several routes. Low-intensity laser irradiation increases blood microcirculation via sympathetic neural inhibition and prompts an increase in cell proliferation and division, thus speeding up the repair of damaged cells in the auditory system [7,8]. Additionally, another study noted that low-power laser stimulation is able to promote ATP synthesis in mitochondria by stimulating glucose combustion in the mitochondria, thus increasing the ATP supply for cell processes and decreasing cell damage [6]. Despite many studies that demonstrated a positive effect of laser irradiation on tinnitus, a previous study has shown that the transmission of light across the tympanic cavity and the promontory depends strongly on several factors. When irradiating the tympanic membrane or mastoid process, the transmitted light crosses anatomic structures in the middle ear, such as the eardrum, auditory ossicles, oval window, temporal bone and promontory bone, which may cause attenuation of laser irradiation. When different parts of the tympanic membrane or mastoid area are illuminated, a different light distribution within the cochlea results. Additionally, increasing the distance between the irradiation fiber tip and the irradiation target leads to attenuation of the transmitted irradiance [15,16]. Therefore, when laser irradiation is administered, the unique anatomy of the ear must be considered, as it may affect the penetration of the irradiance. In a retrospective study [33], a narrowed external acoustic meatus was found in up to 35% of normal people, which means that laser irradiance would be absorbed by the external acoustic meatus before reaching the cochlea. It is difficult to individualize laser treatments according to patients’ anatomical differences, which in turn makes it difficult to obtain the effect of laser irradiation since the majority of studies delivered irradiation transmeatally. Failure to perform controlled and constant irradiation may therefore represent a significant contributing factor to the widely varying therapeutic outcomes in existing studies of LLLT. We suggest more precise and constant delivery by techniques that can overcome the complexity of outer and middle ear structures in future studies to obtain the potential effect of LLLT.

Although some forms of tinnitus are most likely generated in the inner ear by abnormal activity of cochlear hair cells or by dysfunctions of the peripheral part of the auditory nerve, the central nervous system also accounts for some form of subjective tinnitus [4]. Several studies have indicated that the function of nuclei in the ascending auditory pathways is altered in those with tinnitus. Meanwhile, redirection of information to regions of the CNS that do not usually receive certain sources of auditory input occurs in those with tinnitus [34,35,36,37,38,39,40]. In one study that used voxel-based morphometry and functional magnetic resonance imaging to evaluate tinnitus-related functional and anatomical anomalies in the auditory and limbic systems, hyperactivity was present in the primary auditory cortices, posterior auditory cortices and nucleus accumbens of tinnitus patients [3]. Tinnitus associated with SNHL and idiopathic etiology has a certain degree of association with the CNS [4,41] and thus is unlikely to be eliminated by low-level laser irradiation, which affects mainly the peripheral components of the auditory system; these associations further explain the negative result in the subgroup analysis in the present study.

Among the included studies, Mollasadeghi et al. [32] was the only study that enrolled patients with tinnitus caused by noise-induced hearing loss and used transmastoid laser irradiation [32]. Although a previous study considered the transmastoidal route of laser irradiation to be therapeutically insufficient [15], the study demonstrated positive results for LLLT when delivered transmastoidally. As we discussed in the previous paragraph, LLLT might mainly affect the cochlea, and studies have shown that noise trauma can result in injury to hair cells in the inner ear and degeneration of the auditory nerve [42,43]. In this study, the patients who sustained injury from noise trauma to the cochlea might benefit the most from irradiation and therefore show the efficacy of irradiation for tinnitus. Nevertheless, further RCTs are required to support the effect of LLLT on tinnitus caused mainly by specific inner ear disease or injury.

Many studies were excluded due to lack of placebo control in the present meta-analysis. In those single-arm studies, a high percentage of improvement was reported. The favorable outcomes may come from the placebo effects. In one of the excluded studies showing a favorable effect of LLLT, patients also suffered from the comorbidity with of temporal-mandibular joint disorder (TMD) [44], which could benefit from LLLT too [45]. As somatosensory diseases like TMD [46,47,48], chronic headache [49], trigeminal neuralgia [50] or cervical spondylosis [51], could lead to tinnitus and the relief of those somatosensory diseases could also lead to improvement of tinnitus, the relationship between comorbidity and effect of LLLT would serve as the potential bias. To avoid the bias, it was worth emphasizing the importance on patient selection. In the present study, patients were composed of comorbidity of SNHL or idiopathic tinnitus and thus limited the potential bias, which would have been obtained in those excluded studies with comorbidity susceptible to LLLT.

Among the investigated studies, only one patient was reported to have developed sudden onset hearing impairment, and one patient developed dizziness during the course of LLLT [24]. No adverse effects after irradiation were reported or observed in other studies. Meta-analysis was not performed due to the lack of adequate effect estimates in the studies. We still consider the two sporadic adverse effects important, and these effects should be explained when obtaining informed consent from the patient before delivering the laser treatment. We look forward to further studies that analyze the safety of LLLT.

Limitations were present in our study. First, different measurement methods were used in the included studies, including THI and various rating scales (VAS, VRS and NRS). Among studies that report effect estimates with rating scales, converting the original score to the same effect estimate is not feasible. We therefore extracted dichotomous data from studies using the corresponding rating scale. At the same time, we did not stratify the degree of improvement, as some of the included studies did [24,32]. In the present extraction and adjustment, we obtained expanded effect estimates but may have overestimated the efficacy of LLLT. As a result, no significant difference in pooled effect estimates of the rating scale scores is seen, as in the pooled effect estimates of the THI score. Second, heterogeneity exists among the studies in terms of the technical parameters used. Tauber et al. [15] showed that the wavelength of the laser strongly influences the transmission of irradiation to the cochlea since a longer wavelength induces more transmission of the irradiation. The use of different wavelengths among the studies may have influenced cochlear irradiance and the effects of LLLT. Subgroup analysis on the influence of wavelength showed no difference between the two groups, as an identical overall effect was observed. Third, the timing of the effect estimates was examined in the studies. In Mirvakili et al. [26] and Mollasadeghi et al. [32], the treatment effects weakened over time. To minimize the effect of timing and obtain maximal LLLT efficacy, we pooled the effect estimate measured at the earliest timepoint after the intervention. No favorable result was demonstrated despite the adjustment. Finally, different numbers of irradiation sessions were addressed among various studies in this meta-analysis. The median treatment lasted 20 sessions, and we conducted subgroup analysis according to the effect estimate of those who underwent fewer than 20 sessions and those who underwent more than 20 sessions. No significant improvement has been shown with more irradiation sessions. Despite the subgroup analysis comparing the effects under different settings, there are still a number of factors that we were unable to analyze (e.g., different machines, races), and the existing heterogeneity underpowered the analysis. Meanwhile, the relatively small number of patients and studies might further underestimate the potential effect of LLLT, as we may glimpse a better but nonsignificant effect of LLLT in all the analyses. From these perspectives, we suggest that further large-scale, detailed RCTs are essential.

## 5. Conclusions

We evaluated the efficacy of LLLT in patients with tinnitus by meta-analysis. The results showed no favorable effect of LLLT, regardless of the measurement method and technical parameters. We suggest further large-scale studies to evaluate the efficacy of the therapy on tinnitus.

## Figures and Tables

**Table 1:** Study characteristics.

Study	Location	Laser Modality	Sample Size	Mean Age in Treatment Group	Mean Age in Control Group	Comorbidity	Tinnitus Duration (Month)	Control	Irradiation Session	Treatment Laterality	Post-Intervention Measurement		Reported Adverse Events
											Scale	Timing	
Choi et al., 2019 [1]	Korea	100 mW; 830 nm; 20 min/day for 10 sessions	38	53.3	58.4	SNHL	>3 M	Sham laser	Fewer irradiation sessions	Unilateral	THI-total	Immediately	No AEs observed
Dehkordi et al., 2015 [25]	Iran	5 mV; 650 nm; 20 min/day for 20 sessions	66	52.5	46.8	Idiopathic	Treatment group: 48 M Control group: 34 M	Sham laser	More irradiation sessions	Unilateral	NRS-loudness	Immediately	NR
Mirvakili et al., 2014 [26]	Iran	5 mW; 650 nm; 20 min/day for 20 sessions	120	41.08	39.43	SNHL	>12 M	Sham laser	More irradiation sessions	NR	VAS-loudness	Immediately	NR
Nago et al., 2014 [27]	Malaysia	5 mW; 650 nm; 20 min/day for 70 sessions	43	56.5	58.7	Idiopathic	>6 M	Sham laser	More irradiation sessions	NR	VAS-loudness	Immediately	NR
Mollasadeghi et al., 2013 [32]	Iran	5 mW; 650 nm; 20 min/day for 20 sessions	82	41.17		SNHL (NIHL)	22 M (average)	Sham laser	More irradiation sessions	NR	VAS-loudness	Immediately	NR
Teggi et al., 2009 [28]	Italy	5 mW; 650 nm; 20 min/day for 90 sessions	54	51.6	53.1	SNHL	Treatment group: 26 M Control group: 26 M	Sham laser	More irradiation sessions	Unilateral	THI-total	Immediately	NR
Cuda et al., 2008 [29]	Italy	5 mW; 650 nm; 20 min/day for 90 sessions	46	50.3	64.4	Mixed (84.8% HL; 15.2% idiopathic)	>36 M	Sham laser	More irradiation sessions	NR	THI-total	Immediately	NR
Gungor et al., 2008 [23]	Turkey	5 mW; 650 nm; 15 min/day for 7 sessions	66 *	55.8		Mixed (54% HL; 45% idiopathic)	96 M (average)	Sham laser	Fewer irradiation sessions	Unilateral	VRS-loudness	2 weeks after treatment	No AEs observed
Rhee et al., 2006 [30]	Korea	67 mW; 830 nm; 20 min/day for 12 sessions	50	49.2	52.3	Idiopathic	Treatment group: 17 M Control group: 20 M	NR	Fewer irradiation sessions	NR	THI-total	1 week after treatment	No AEs observed
Nakashima et al., 2002 [24]	Japan	60 mW; 810 nm; 6 min per week for 4 sessions	64 *	52.4	55.2	SNHL	NR	Sham laser	Fewer irradiation sessions	NR	VRS-loudness	1 week after treatment	Sudden deafness and dizziness
Mirz et al., 1999 [31]	Denmark	50 mW; 830 nm; 15 min/time for 15 sessions	41	48.6	48.7	Idiopathic	Treatment group: 70 M Control group: 62 M	Sham laser	Fewer irradiation sessions	Unilateral	THI-total	Immediately	NR

SNHL: Sensorineural hearing loss; NIHL: Noise-induced hearing loss; THI: Tinnitus Handicap Inventory; NRS: Numeric rating scale; VRS: Verbal rating scale NR: Not reported. Studies marked with asterisks used ears as the study subjects; M: months.

## Supplementary Materials

The following are available online at https://www.mdpi.com/2076-3425/10/12/931/s1, Table S1: Detailed search strategy, Figure S1: Risk of Bias, Figure S2: Influence analysis of studies with THI measurement, Figure S3: Influence analysis of studies with improvement rate by rating scale, Figure S4: TSA for LLLT on overall THI, Figure S5: TSA for LLLT on THI with SNHL, Figure S6: TSA for LLLT on THI with idiopathic tinnitus, Figure S7: TSA for LLLT on THI with more irradiation, Figure S8: TSA for LLLT on THI with less irradiation, Figure S9: TSA for LLLT on THI with 650 nm wavelength, Figure S10: TSA for LLLT on THI with 830 nm wavelength, Figure S11: TSA for LLLT on overall improvement rate, Figure S12: TSA for LLLT on improvement rate with SNHL, Figure S13: TSA for LLLT on improvement rate with idiopathic tinnitus, Figure S14: TSA for LLLT on improvement rate with more irradiation, Figure S15: TSA for LLLT on improvement rate with less irradiation.
