# Supplementary material for: Efficacy of Low-Level Laser Therapy for Tinnitus: A Systematic Review with Meta-Analysis and Trial Sequential Analysis
Source: Brain Sci. 2020 Dec 2;10(12):931. doi: 10.3390/brainsci10120931 (PMC7761002; doi:10.3390/brainsci10120931)
Supplement: Supplementary file 1 [file brainsci-10-00931-s001.pdf]

Supplemental Materials

Table S1. Detailed search strategy.

Database	Query
PubMed	("Tinnitus"[Mesh] OR "Tinnitus/complications"[Mesh] OR "Tinnitus/diagnosis"[Mesh] OR "Tinnitus/prevention and control"[Mesh] OR "Tinnitus/therapy"[Mesh] OR "Tinnitus"[TIAB] OR "Pulsatile Tinnitus"[TIAB] OR "Clicking Tinnitus"[TIAB] OR "Noise Induced Tinnitus"[TIAB] OR "Objective Tinnitus"[TIAB] OR "Subjective Tinnitus"[TIAB] OR "Tinnitus of Vascular Origin"[TIAB] OR "Tinnitus" OR "Pulsatile Tinnitus" OR "Clicking Tinnitus" OR "Noise Induced Tinnitus" OR "Objective Tinnitus" OR "Subjective Tinnitus" OR "Tinnitus of Vascular Origin") AND ("Lasers"[Mesh] OR "Lasers/instrumentation"[Mesh] OR "Lasers/methods"[Mesh] OR "Lasers/pharmacology"[Mesh] OR "Lasers/therapeutic use"[Mesh] OR "Laser"[TIAB] OR "Lasers"[TIAB] OR "Q-Switched Lasers"[TIAB] OR "Q Switched Lasers"[TIAB] OR "Q-Switched Laser"[TIAB] OR "Pulsed Lasers"[TIAB] OR "Pulsed Laser"[TIAB] OR "Continuous Wave Lasers"[TIAB] OR "Continuous Wave Laser"[TIAB] OR "Masers"[TIAB] OR "Maser"[TIAB] OR "Laser" OR "Lasers" OR "Q-Switched Lasers" OR "Q Switched Lasers" OR "Q-Switched Laser" OR "Pulsed Lasers" OR "Pulsed Laser" OR "Continuous Wave Lasers" OR "Continuous Wave Laser" OR "Masers" OR "Maser")
Embase	('tinnitus'/exp OR tinnitus OR 'pulsatile tinnitus'/exp OR 'pulsatile tinnitus' OR 'clicking tinnitus' OR 'noise induced tinnitus' OR 'objective tinnitus' OR 'subjective tinnitus' OR 'tinnitus of vascular origin') AND ('laser'/exp OR laser OR lasers OR 'q-switched lasers' OR 'q switched lasers' OR 'q-switched laser' OR 'pulsed lasers' OR 'pulsed laser'/exp OR 'pulsed laser' OR 'continuous wave lasers' OR 'continuous wave laser'/exp OR 'continuous wave laser' OR masers OR maser) AND ('placebo'/exp OR placebo)
Web of Science	TS=(("Tinnitus" OR "Pulsatile Tinnitus" OR "Clicking Tinnitus" OR "Noise Induced Tinnitus" OR "Objective Tinnitus" OR "Subjective Tinnitus" OR "Tinnitus of Vascular Origin")AND("Laser" OR "Lasers" OR "Q-Switched Lasers" OR "Q Switched Lasers" OR "Q-Switched Laser" OR "Pulsed Lasers" OR "Pulsed Laser" OR "Continuous Wave Lasers" OR "Continuous Wave Laser" OR "Masers" OR "Maser"))
Scopus	(TITLE-ABS-KEY("Tinnitus" OR "Pulsatile Tinnitus" OR "Clicking Tinnitus" OR "Noise Induced Tinnitus" OR "Objective Tinnitus" OR "Subjective Tinnitus" OR "Tinnitus of Vascular Origin") AND TITLE-ABS-KEY("Laser" OR "Lasers" OR "Q-Switched Lasers" OR "Q Switched Lasers" OR "Q-Switched Laser" OR "Pulsed Lasers" OR "Pulsed Laser" OR "Continuous Wave Lasers" OR "Continuous Wave Laser" OR "Masers" OR "Maser"))
Cochrane Library	ID SearchHits<br>ID Search<br>#1 MeSH descriptor: [Tinnitus] explode all trees<br>#2 MeSH descriptor: [Tinnitus] explode all trees and with qualifier(s): [complications - CO]<br>#3 MeSH descriptor: [Tinnitus] explode all trees and with qualifier(s): [diagnosis - DI]<br>#4 MeSH descriptor: [Tinnitus] explode all trees and with qualifier(s): [prevention & control - PC]<br>#5 MeSH descriptor: [Tinnitus] explode all trees and with qualifier(s): [therapy - TH]<br>#6 Tinnitus<br>#7 Pulsatile Tinnitus<br>#8 Clicking Tinnitus<br>#9 Noise Induced Tinnitus<br>#10 Objective Tinnitus<br>#11 Subjective Tinnitus<br>#12 Tinnitus of Vascular Origin<br>#13 {OR #1-#12}<br>#14 MeSH descriptor: [Lasers] explode all trees<br>#15 Laser<br>#16 Lasers<br>#17 Q-Switched Laser<br>#18 Q-Switched Lasers<br>#19 Q Switched Lasers<br>#20 Pulsed Lasers<br>#21 Pulsed Laser<br>#22 Continuous Wave Lasers<br>#23 Continuous Wave Laser<br>#24 Masers<br>#25 Maser<br>#26 {OR #14-#25}<br>#27 #13 AND #26

Studies with intention-to-treat	Unique ID	Study ID	Experimental	Comparator	Outcome	Weight	Randomization process	Deviations from intended interven	Missing outcome data	Measurement of the outcome	Selection of the reported result	Overall
	Choi et al., 2019	1	Low-level laser	placebo	Improvement of tinnitus	1	+	+	+	?	+	+
	Dehkordi et al., 2015	6	Low-level laser	placebo	Improvement of tinnitus	1	+	+	+	?	+	!
	Mirvakili et al., 2014	62	Low-level laser	placebo	Inprovement of tinnitus	1	?	+	+	?	+	!
	Nago et al., 2013	8	Low-level laser	placebo	Inprovement of tinnitus	1	+	+	+	?	+	!
	Mollasadeghi al., 2013	13	Low-level laser	placebo	Inprovement of tinnitus	1	+	+	+	?	+	!
	Teggi et al., 2009	45	Low-level laser	placebo	Inprovement of tinnitus	1	+	+	+	+	+	+
	Cuda et al., 2008	16	Low-level laser	placebo	Inprovement of tinnitus	1	+	+	+	?	+	!
	Gungor et al., 2008	17	Low-level laser	placebo	Inprovement of tinnitus	1	+	+	+	?	+	!
	Rhee et al., 2006	269	Low-level laser	placebo	Inprovement of tinnitus	1	+	+	+	?	+	!
	Nakashima et al., 2002	46	Low-level laser	placebo	Inprovement of tinnitus	1	+	+	+	?	+	!
	Mirz et al., 1999	28	Low-level laser	placebo	Inprovement of tinnitus	1	+	+	+	?	+	!

+ Low risk
? Some concerns
? High risk

Figure S1. Risk of Bias. The revised Cochrane Risk of Bias Tool 2 was used to evaluate to quality of included studies [1–11].

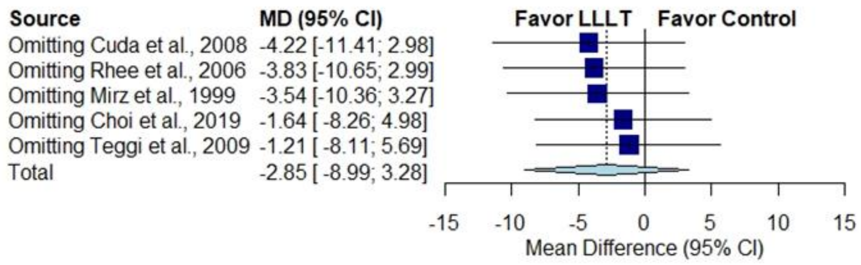

Figure S2. Influence analysis of studies with THI measurement. The pooled point estimates after excluding every study one by one were contained within the 95% CI of the overall pooled results for these outcomes. No extreme effect sizes(outlier) was identified [1,3,4,6,11].

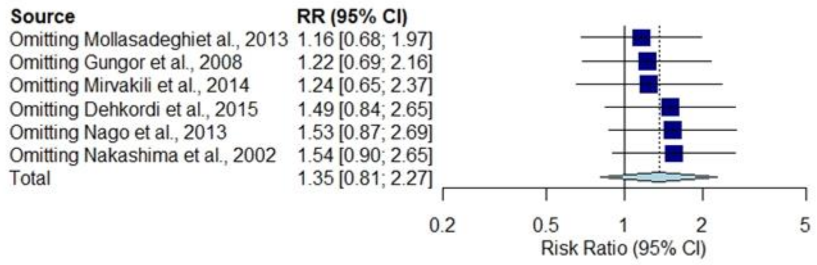

Figure S3. Influence analysis of studies with improvement rate by rating scale. The pooled point estimates after excluding every study one by one were contained within the 95% CI of the overall pooled results for these outcomes. No extreme effect sizes(outlier) was identified [2,5,7–10].

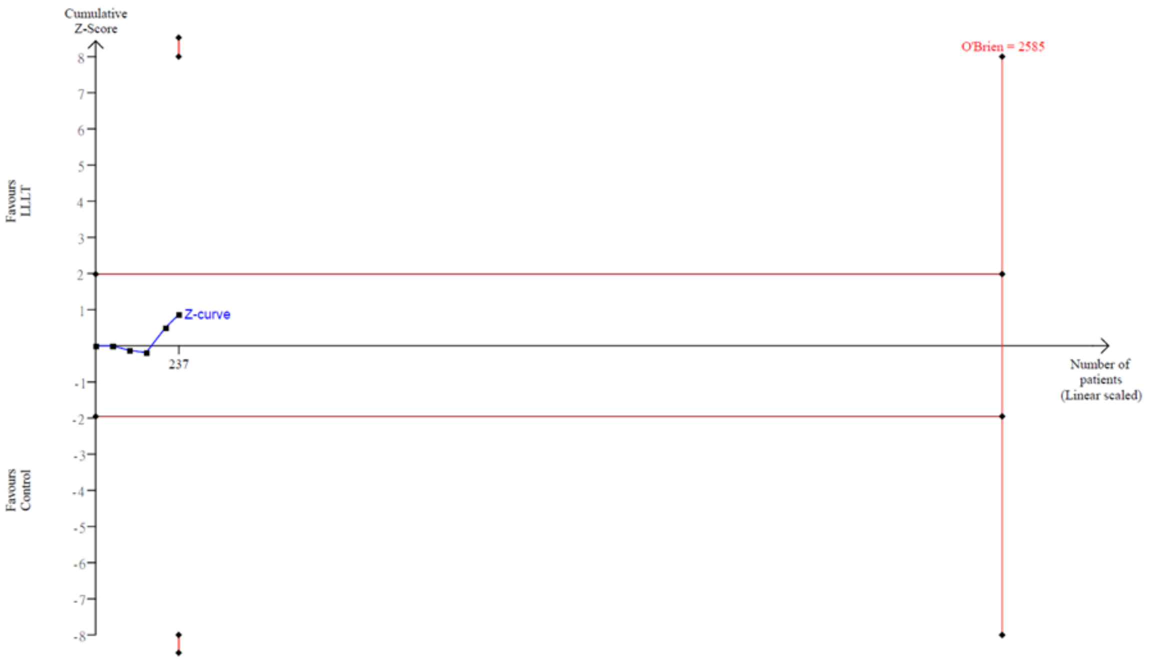

Figure S4. TSA for LLLT on overall THI. The cumulative Z-curves didn't surpass the traditional significance boundary or the sequential monitoring boundaries for the adjusted significance threshold in favor of LLLT.

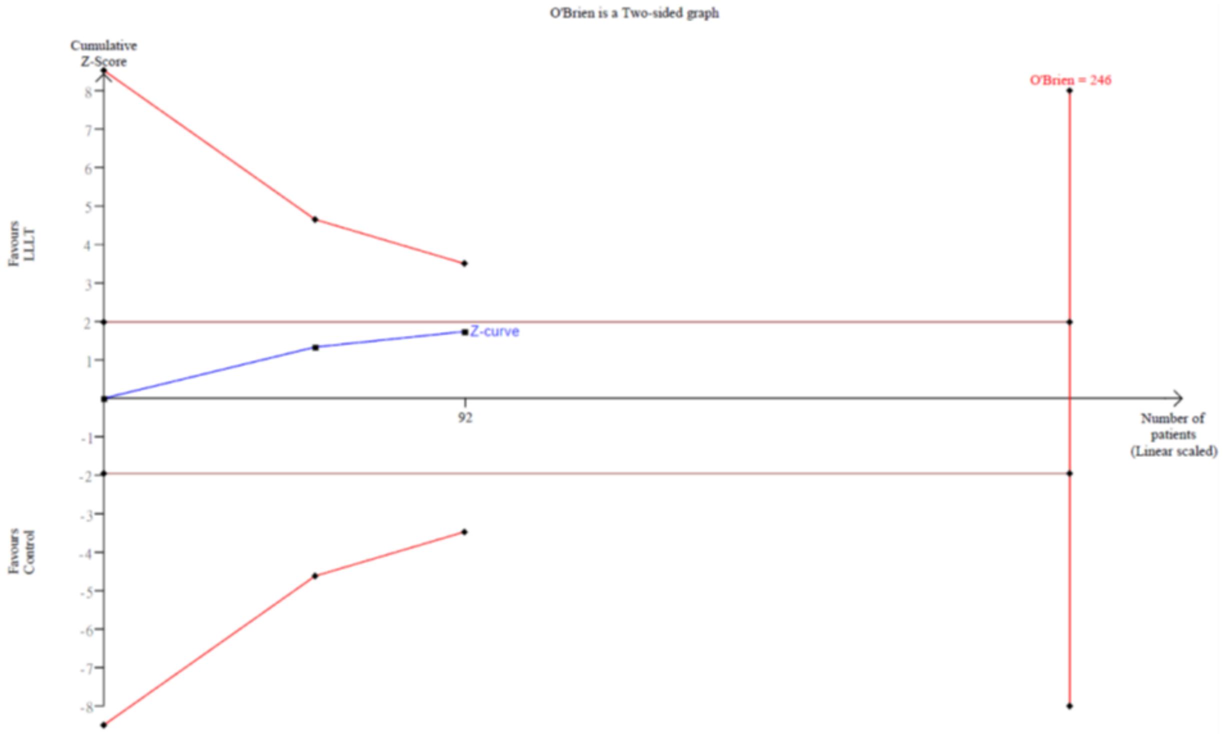

Figure S5. TSA for LLLT on THI with SNHL. The cumulative Z-curves didn't surpass the traditional significance boundary or the sequential monitoring boundaries for the adjusted significance threshold in favor of LLLT.

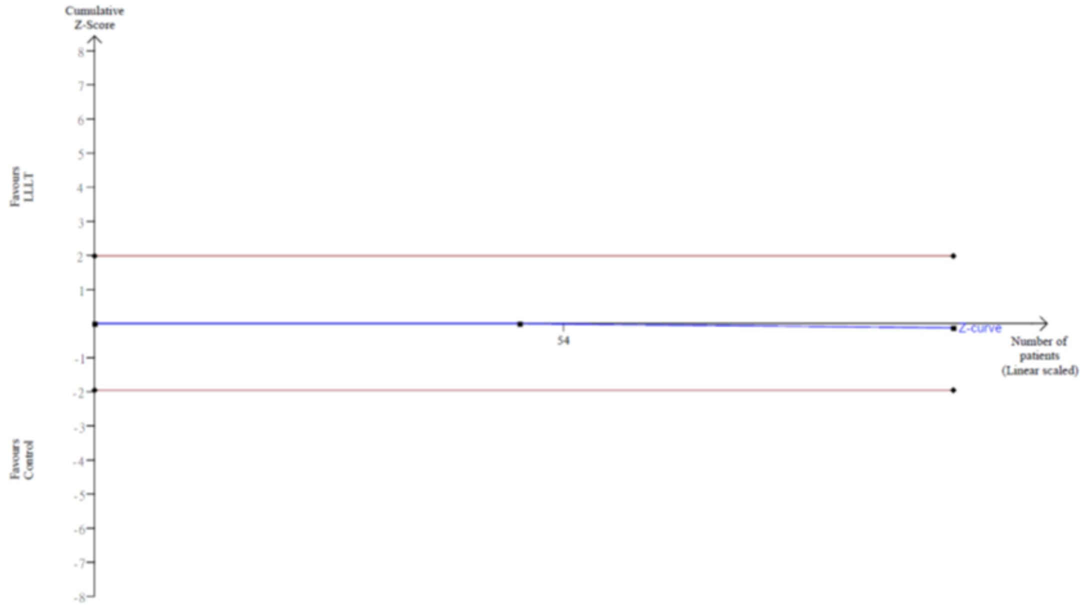

Figure S6. TSA for LLLT on THI with idiopathic tinnitus. Boundary was ignored due to little information use (0.23%). The cumulative Z-curves didn't surpass the traditional significance boundary in favor of LLLT.

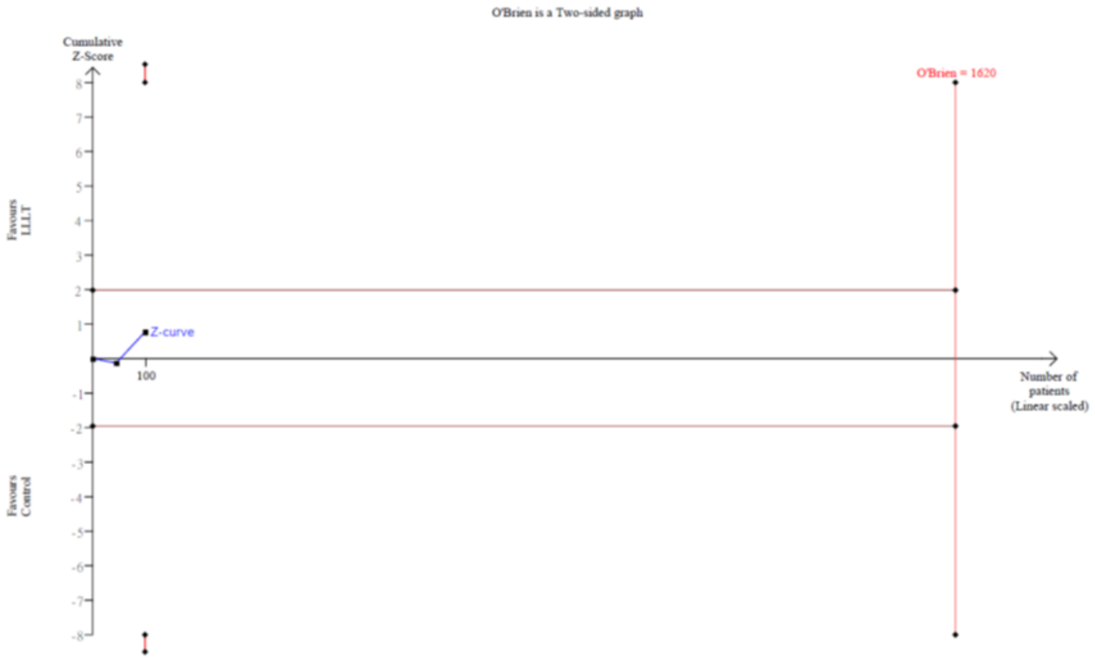

Figure S7. TSA for LLLT on THI with more irradiation. The cumulative Z-curves didn't surpass the traditional significance boundary or the sequential monitoring boundaries for the adjusted significance threshold in favor of LLLT.

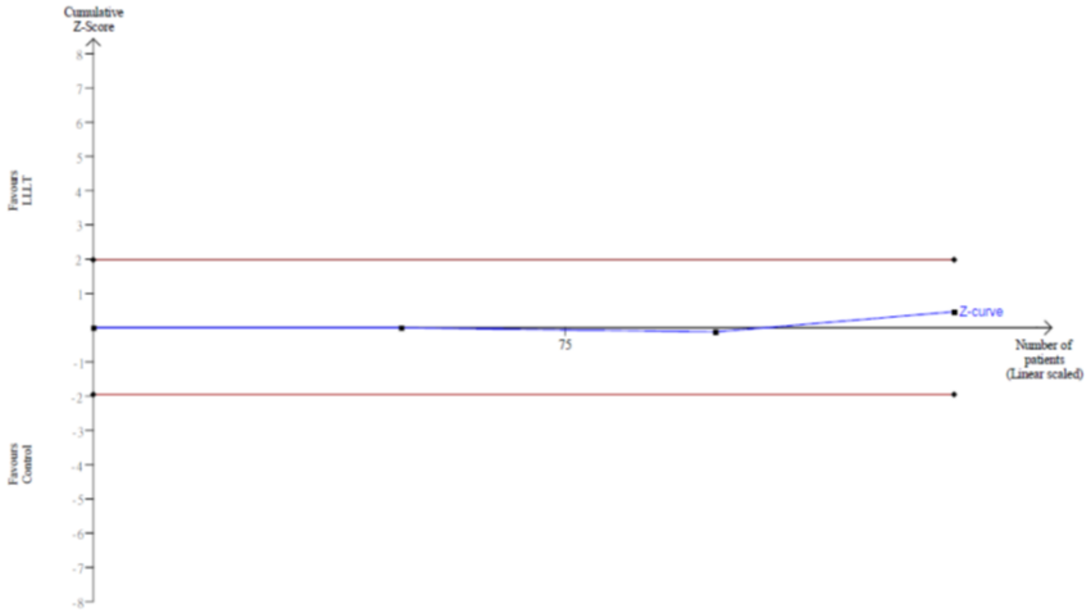

Figure S8. TSA for LLLT on THI with less irradiation. Boundary was ignored due to little information use (2.6%). The cumulative Z-curves didn't surpass the traditional significance boundary in favor of LLLT.

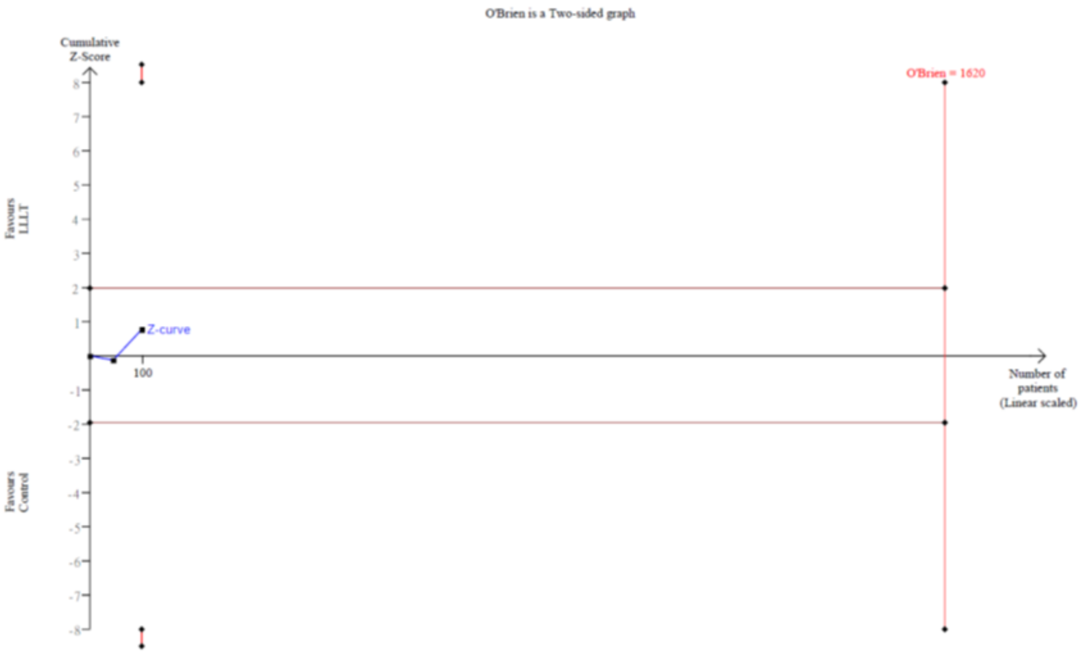

Figure S9. TSA for LLLT on THI with 650 nm wavelength. The cumulative Z-curves didn't surpass the traditional significance boundary or the sequential monitoring boundaries for the adjusted significance threshold in favor of LLLT.

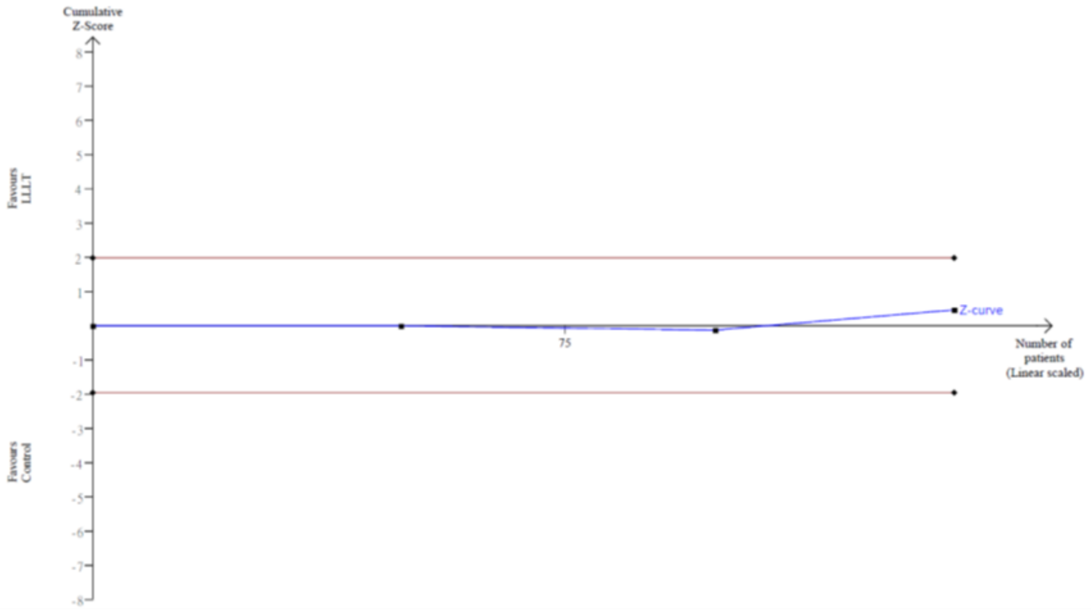

Figure S10. TSA for LLLT on THI with 830 nm wavelength. Boundary was ignored due to little information use (2.6%). The cumulative Z-curves didn't surpass the traditional significance boundary in favor of LLLT.

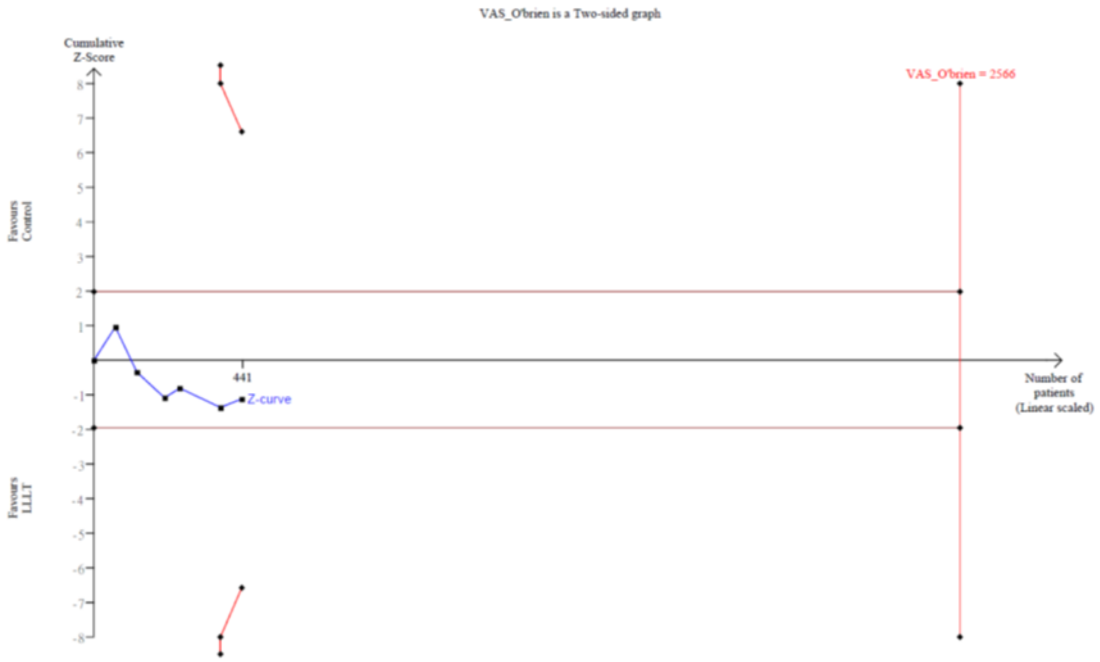

Figure S11. TSA for LLLT on overall improvement rate. The cumulative Z-curves didn't surpass the traditional significance boundary or the sequential monitoring boundaries for the adjusted significance threshold in favor of LLLT.

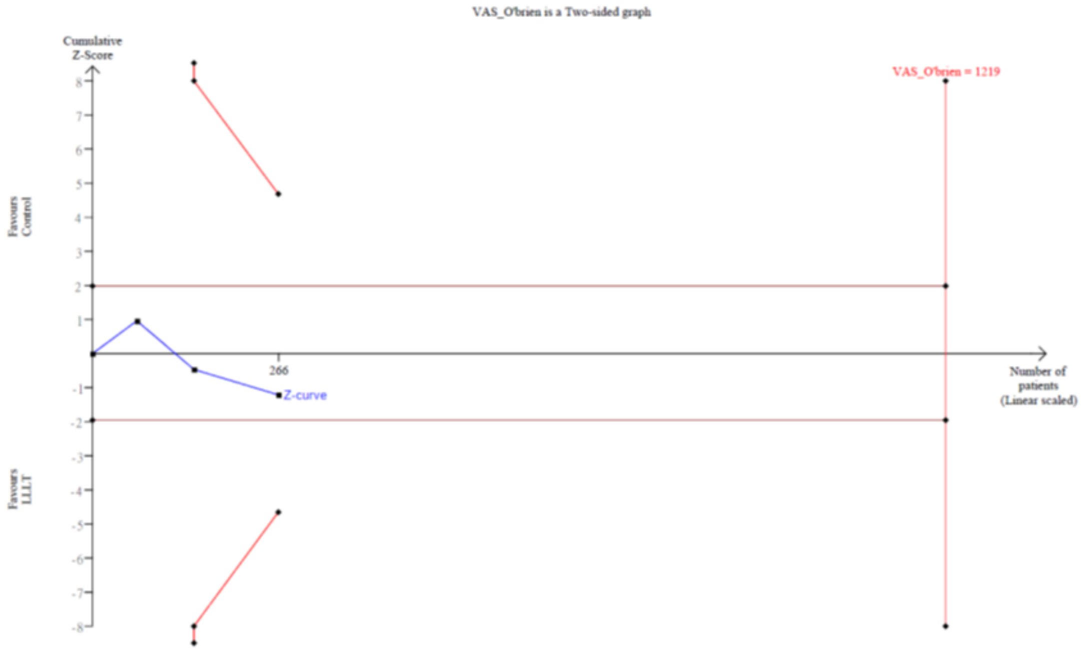

Figure S12. TSA for LLLT on improvement rate with SNHL. The cumulative Z-curves didn't surpass the traditional significance boundary or the sequential monitoring boundaries for the adjusted significance threshold in favor of LLLT.

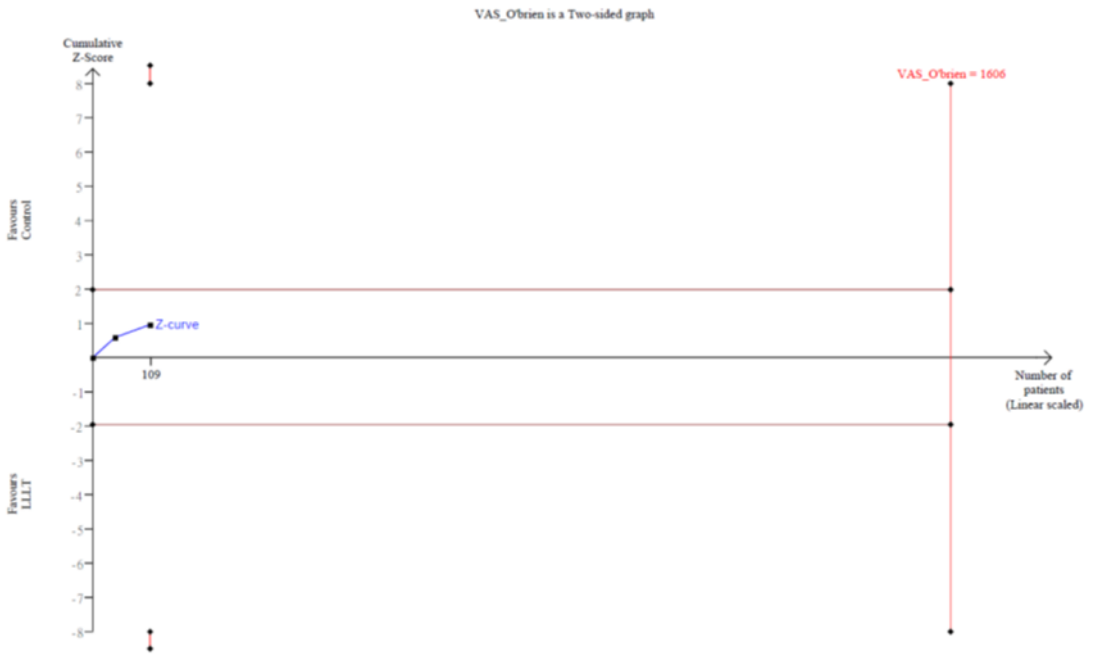

Figure S13. TSA for LLLT on improvement rate with idiopathic tinnitus. The cumulative Z-curves didn't surpass the traditional significance boundary or the sequential monitoring boundaries for the adjusted significance threshold in favor of LLLT.

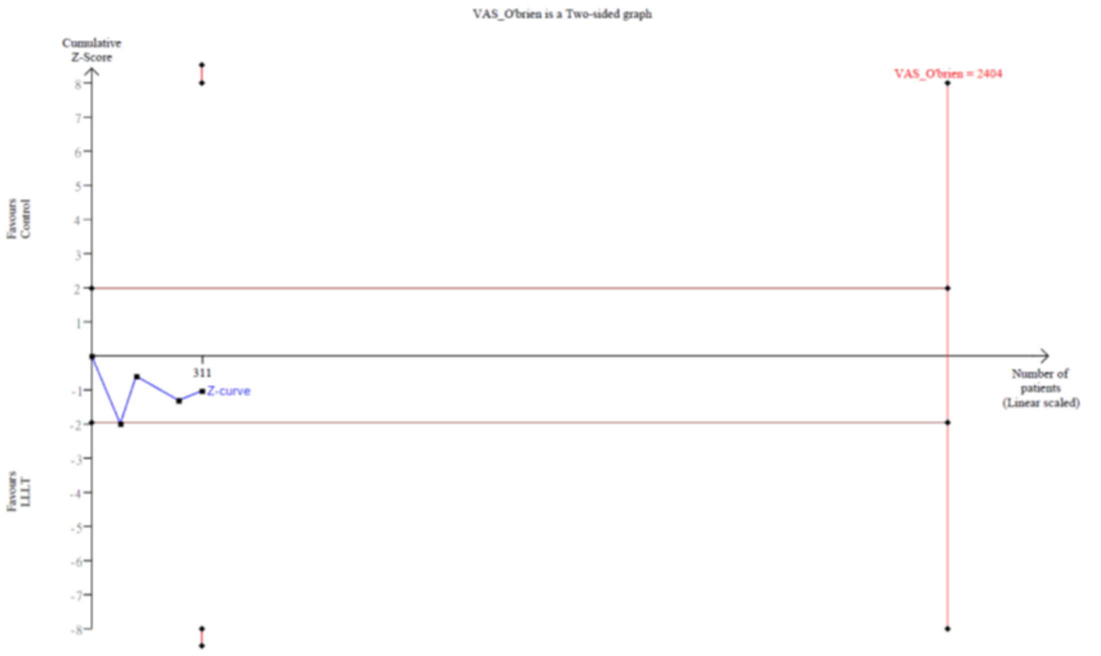

Figure S14. TSA for LLLT on improvement rate with more irradiation. The cumulative Z-curves didn't surpass the traditional significance boundary or the sequential monitoring boundaries for the adjusted significance threshold in favor of LLLT.

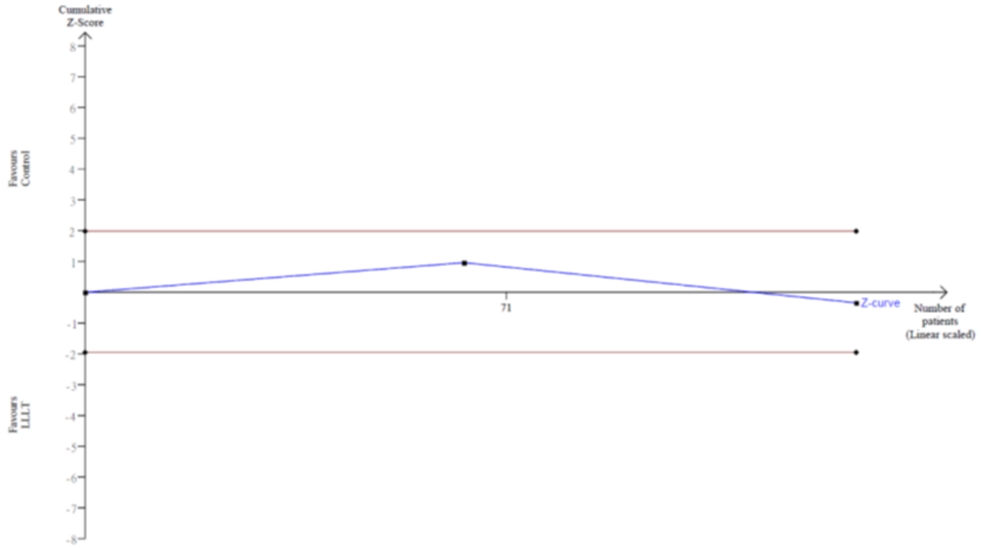

Figure S15. TSA for LLLT on improvement rate with less irradiation. The cumulative Z-curves didn't surpass the traditional significance boundary or the sequential monitoring boundaries for the adjusted significance threshold in favor of LLLT.

References

1. Mirz, F.; Zachariae, R.; Andersen, S.E.; Nielsen, A.G.; Johansen, L.V.; Bjerring, P.; Pedersen, C.B. The low-power laser in the treatment of tinnitus. *Clinical Otolaryngology and Allied Sciences* **1999**, *24*, 346–354, doi:10.1046/j.1365-2273.1999.00277.x.
2. Nakashima, T.; Ueda, H.; Misawa, H.; Suzuki, T.; Tominaga, M.; Ito, A.; Numata, S.; Kasai, S.; Asahi, K.; Vernon, J.A., et al. Transmeatal low-power laser irradiation for tinnitus. *Otology & neurotology* **2002**, *23*, 296-300, doi:10.1097/00129492-200205000-00011.
3. Rhee, C.K.; Lim, E.S.; Kim, Y.S.; Chung, Y.W.; Jung, J.Y.; Chung, P.S. Effect of low level laser (LLL) on cochlear and vestibular inner ear including tinnitus.
4. Cuda, D.; De Caria, A. Effectiveness of combined counseling and low-level laser stimulation in the treatment of disturbing chronic tinnitus. *International Tinnitus Journal* **2008**, *14*, 175–180.
5. Gungor, A.; Dogru, S.; Cincik, H.; Erkul, E.; Poyrazoglu, E. Effectiveness of transmeatal low power laser irradiation for chronic tinnitus. *Journal of Laryngology and Otology* **2008**, *122*, 447–451, doi:10.1017/S0022215107009619.
6. Teggi, R.; Bellini, C.; Piccioni, L.O.; Palonta, F.; Bussi, M. Transmeatal low-level laser therapy for chronic tinnitus with cochlear dysfunction. *Audiology & neuro-otology* **2009**, *14*, 115-120, doi:10.1159/000161235.
7. Mollasadeghi, A.; Mirmohammadi, S.J.; Mehrparvar, A.H.; Davari, M.H.; Shokouh, P.; Mostaghaci, M.; Baradaranfar, M.H.; Bahaloo, M. Efficacy of low-level laser therapy in the management of tinnitus due to noise-induced hearing loss: A double-blind randomized clinical trial. *The Scientific World Journal* **2013**, *2013*, doi:10.1155/2013/596076.
8. Mirvakili, A.; Mehrparvar, A.; Mostaghaci, M.; Mollasadeghi, A.; Mirvakili, M.; Baradaranfar, M. Low level laser effect in treatment of patients with intractable tinnitus due to sensorineural hearing loss. *Journal of lasers in medical sciences* **2014**, *5*, 71-74.
9. Ngao, C.F.; Tan, T.S.; Narayanan, P.; Raman, R. The effectiveness of transmeatal low-power laser stimulation in treating tinnitus. *European Archives of Oto-Rhino-Laryngology* **2014**, *271*, 975–980, doi:10.1007/s00405-013-2491-3.
10. Dehkordi, M.A.; Einolghozati, S.; Ghasemi, S.M.; Abolbashari, S.; Meshkat, M.; Behzad, H. Effect of low-level laser therapy in the treatment of cochlear tinnitus: A double-blind, placebo-controlled study. *Ear, Nose and Throat Journal* **2015**, *94*, 32–36.
11. Choi, J.E.; Lee, M.Y.; Chung, P.S.; Jung, J.Y. A preliminary study on the efficacy and safety of low level light therapy in the management of cochlear tinnitus: A single blind randomized clinical trial. *International Tinnitus Journal* **2019**, *23*, 52–57, doi:10.5935/0946-5448.20190010.
